# P-464. Challenges of Microbiological Diagnosis and Targeted Treatment of Pediatric Sinogenic Intracranial Infections

**DOI:** 10.1093/ofid/ofaf695.679

**Published:** 2026-01-11

**Authors:** Taylor Boyd, Nicole L Pershing, Isabella F McNamara, Jared Olson, Anne J Blaschke

**Affiliations:** Spencer Fox Eccles School of Medicine, University of Utah, Salt Lake, UT; University of Utah School of Medicine, Salt Lake City, Utah; Tufts Medicine, Boston, Massachusetts; Intermountain Health, Salt Lake City, Utah; University of Utah School of Medicine, Salt Lake City, Utah

## Abstract

**Background:**

Intracranial extension of infectious sinusitis is a rare but potentially life-threatening childhood disease with increasing incidence. *Streptococcus anginosus* group (SAG) species are the most frequently identified pathogens, but infections are often polymicrobial. Current guidelines do not address potential use of ertapenem for this infection.

**Methods:**

We performed a retrospective chart review of patients admitted to Primary Children’s Hospital with ICD9 and ICD10 diagnoses of sinusitis and intracranial infection between 2004 and 2019. We compared microbiologic data from surgical cultures by anatomic site, and course and outcomes stratified by culture-guided antibiotic treatment.

**Results:**

We evaluated 85 sinogenic intracranial infections. Most patients (n=81) underwent surgical debridement. Surgical cultures from the sinus (n=62) and central nervous system (CNS, n=67) were reviewed. Multiple bacteria were identified in 62% of cases. SAG were the most commonly identified pathogen (n=44). Most patients had surgical cultures from the sinus and CNS (n=48). Microbiologic results were discordant between sites in 83% of cases, including Staphylococcus aureus detected only in sinus cultures (n=12) and SAG detected only from CNS cultures (n=10). Anaerobic organisms were identified in 32% of patients overall, but discordant for 60% of patients with both cultures. CNS culture-guided antibiotics included ceftriaxone/metronidazole (n=36), ertapenem (n=22), meropenem (n=11), and other combinations (n=16). Neurologic complications persisting at 1-year were rare and included epilepsy and hemiparesis. There were no statistically significant differences in surgical management or chronic neurologic complications between treatment groups.

**Conclusion:**

Sinogenic intracranial infections are typically polymicrobial including SAG and anaerobes. Timely CNS surgical cultures are important to guide treatment. Sinus culture data may not accurately reflect CNS pathogens. Culture directed antibiotic treatment with ceftriaxone/metronidazole and carbapenems were equally effective with similarly low rates of long-term neurologic sequelae. Additional data is needed to inform generalizability of these findings.

**Disclosures:**

Anne J. Blaschke, MD, PhD, BioFire Diagnostics/Biomerieux: I have IP owned by the U. of Utah licensed to BioFire and receive royalties.|Merck & Co: Advisor/Consultant

